# Optimization of a Membrane Feeding Assay for *Plasmodium vivax* Infection in *Anopheles albimanus*

**DOI:** 10.1371/journal.pntd.0004807

**Published:** 2016-06-29

**Authors:** Andrés F. Vallejo, Kelly Rubiano, Andres Amado, Amy R. Krystosik, Sócrates Herrera, Myriam Arévalo-Herrera

**Affiliations:** 1 Malaria Vaccine and Drug Development Center (MVDC), Cali, Valle de Cauca, Colombia; 2 Caucaseco Scientific Research Center, Cali, Cali, Valle de Cauca, Colombia; 3 Kent State University College of Public Health, Kent, Ohio, United States of America; Fundaçao Oswaldo Cruz, BRAZIL

## Abstract

**Introduction:**

Individuals exposed to malaria infections for a long time develop immune responses capable of blocking *Plasmodium* transmission to mosquito vectors, potentially limiting parasite spreading in nature. Development of a malaria TB vaccine requires a better understanding of the mechanisms and main effectors responsible for transmission blocking (TB) responses. The lack of an *in vitro* culture system for *Plasmodium vivax* has been an important drawback for development of a standardized method to assess TB responses to this parasite. This study evaluated host, vector, and parasite factors that may influence *Anopheles* mosquito infection in order to develop an efficient and reliable assay to assess the TB immunity.

**Methods/Principal Findings:**

A total of 94 *P*. *vivax* infected patients were enrolled as parasite donors or subjects of direct mosquito feeding in two malaria endemic regions of Colombia (Tierralta, and Buenaventura). Parasite infectiousness was assessed by membrane feeding assay or direct feeding assay using laboratory reared *Anopheles* mosquitoes. Infection was measured by qPCR and by microscopically examining mosquito midguts at day 7 for the presence of oocysts.

Best infectivity was attained in four day old mosquitoes fed at a density of 100 mosquitos/cage. Membrane feeding assays produced statistically significant better infections than direct feeding assays in parasite donors; cytokine profiles showed increased IFN-γ, TNF and IL-1 levels in non-infectious individuals. Mosquito infections and parasite maturation were more reliably assessed by PCR compared to microscopy.

**Conclusions:**

We evaluated mosquito, parasite and host factors that may affect the outcome of parasite transmission as measured by artificial membrane feeding assays. Results have led us to conclude that: 1) optimal mosquito infectivity occurs with mosquitoes four days after emergence at a cage density of 100; 2) mosquito infectivity is best quantified by PCR as it may be underestimated by microscopy; 3) host cellular immune response did not appear to significantly affect mosquito infectivity; and 4) no statistically significant difference was observed in transmission between mosquitoes directly feeding on humans and artificial membrane feeding assays.

## Author Summary

Here we assessed the host, vector, and parasite factors for membrane feeding assay using *Plasmodium vivax* and *Anopheles albimanus* mosquitoes and samples from endemic regions of Colombia. This membrane feeding assay method allowed more efficient assessment of the presence of TB activity in sera of individuals from malaria endemic regions as well as in sera of immunized animals and humans. This method can contribute to better assess the TB immune responses elicited by natural infection as well as further evaluation of protective efficacy of malaria TB vaccines.

## Introduction

Malaria is transmitted in 97 countries worldwide where a total of ~207 million cases and ~528,000 deaths are reported yearly. *Plasmodium vivax*, the second most prevalent malaria parasite species, is endemic in 58 countries where annually between 16 and 22.2 million cases are reported [1]. Individuals continuously exposed to malaria infections develop clinical immunity that protects them from severe and complicated disease [2]. This immunity and features in the parasite biology i.e. presence of hypnozoites leads to the presence of a significant number of asymptomatic infections in malaria endemic regions [3]; although sterile immunity is never achieved under natural conditions [4]. Individuals in these communities also develop immune responses that reduce or completely block parasite transmission to *Anopheles* mosquitoes in what has been called transmission blocking (TB) immunity [5] which may play an important role in decreasing malaria transmission in endemic areas [6–8].

Both the mechanisms involved in TB activity as well as the biology of *Plasmodium* transmission from human to mosquito are poorly understood [9, 10]. Multiple host, parasite and vector factors appear to be involved in this transmission. Although malaria-causing parasites *P*. *vivax* and *P*. *falciparum* are similar in some features, there are important biological differences between them. *P*. *vivax* is characterized by inducing periodical clinical relapses due to the spontaneous activation of liver hypnozoites, which do not develop in *P*. *falciparum* infections. Additionally, *P*. *falciparum* merozoites invade erythrocytes of all ages, and this species is known to develop gametocytes in a relatively late phase of the erythrocytic cycle, whereas *P*. *vivax* invasion is restricted to reticulocytes and gametocytes appear in blood significantly earlier. It is therefore likely that earlier appearance of *P*. *vivax* gametocytes translates into greater transmissibility[11]. Factors such as total parasite density, gametocyte maturation stage, male/female gametocyte ratio and others may affect mosquito infection outcomes[12, 13]. Furthermore, differences regarding acquisition of immunity to *P*. *falciparum* and *P*. *vivax* can be of great importance for the development of TB immunity. It has been suggested that clinical immunity against *P*. *vivax* is established earlier in life and consequently, it could require fewer malaria episodes to be developed than *P*. *falciparum*[14]. In our studies [15–18], most asymptomatic infections associate with submicroscopic infections, which may be reflecting the low availability of reticulocytes as well as efficient clinical immunity.

Additionally, mosquito factors appear critical e.g. genetic diversity, as only a limited number of anopheline species are competent vectors and not all females of a given species are equally susceptible to infection [19]. Additionally, mosquito characteristics such as longevity, pH, midgut temperature [20, 21] and microbiota are critical for successful infection [22, 23]. Furthermore, human host factors such as the degree of malaria immunity, diet and nutritional status, as well as drug therapies, could influence *Plasmodium* transmission to mosquitoes.

Because of the availability of highly synchronized mature *P*. *falciparum* gametocytes in culture, i.e. NF54 strain, parasite transmission and TB effects can be readily measured in laboratory conditions adding test sera or antimalarial products to gametocyte-enriched cultured blood used to feed laboratory reared mosquitoes [24, 25]. It has been possible to develop a highly standardized mosquito membrane feeding assay (SMFA) which allows artificial mosquito infections to routinely evaluate the ability of naturally elicited antimalarial antibodies to block mosquito infection. Mosquito feeding can be performed either by direct biting on infected malaria patients or by *ex vivo* exposure to infected blood delivered through glass devices covered with an artificial membrane. While the first method requires the availability of infected patients carrying mature gametocytes in blood circulation and the fulfillment of ethical restrictions, the second is based on the use of cultured parasites such as *Plasmodium falciparum*, or in the case of *Plasmodium vivax*, due to the lack of *in vitro* cultures, the use of infected blood directly obtained from patients is required. TB can be assessed based on different parameters such as: recution in the number of infected mosquitoes, reductions in the oocysts counts per mosquito, or by the reduction on sporozoite production.

Mosquito TB assays [SMFA and membrane feeding assay (MFA)] permit epidemiological studies to determine the prevalence of TB activity in endemic communities [24, 25] or to evaluate TB activity of antibodies elicited artificially by vaccination with specific parasite antigens or mosquito components being tested as TB vaccine candidates [26, 27].

Epidemiological studies conducted in Africa, indicate the presence of TB immunity. Likewise studies carried out in Asia [28] and Latin America [29, 30], indicate a high prevalence of *P*. *vivax* TB activity in endemic communities. On the other hand, several *P*. *falciparum* surface antigens have been proposed and tested as TB vaccine candidates. The parasite antigen PfS25 is expressed in oocysts/ookinete that induces complete TB activity and is currently under clinical development [31, 32]. The gametocyte antigen involved in the fertilization process *Pfs48/45*, is also in clinical development [32]. In the case of *P*. *vivax*, TB vaccine development has proved difficult due to technical constraints imposed by the lack of parasite culture methods. For this species the most advanced TB vaccine candidate is *Pvs*25 which has shown induction of high TB activity in preclinical studies and has a clinical product tested in phase 1 trial [27, 33]. However, several *P*. *vivax* vaccine candidates including *Pvs48/45*, *Pvs230* and *Pvs47* are under development [26, 32]

An additional constraint to study *P*.*vivax* TB is the scarcity of laboratory colonies of *P*. *vivax* susceptible *Anopheles* mosquitoes, which limits the studies on *P*. *vivax* TB immunity [30]. *Anopheles albimanus* is widely distributed in the American continent, ranging from southern regions of Mexico to northern Peru. This mosquito species has been found naturally infected by both *P*. *vivax* and *P*. *falciparum* and is considered a primary malaria vector in Latin America. In spite of being less susceptible[34], this mosquito species is easy to colonize, and it has been used for several years under laboratory conditions in different centers for malaria sporozoite production and TB studies[30, 35–37].

This is a preliminary study focused on the optimization of the currently available *P*. *vivax* MFA in *An*. *albimanus* [30] and on comparing controlled MFA and DFA assays in order to further establish optimal conditions for reliably determining the prevalence and intensity of TB activity present in communities naturally exposed to *P*. *vivax* malaria. Furthermore, an optimized *P*. *vivax* MFA could be employed to assess the responses elicited by vaccination with *P*. *vivax* TB vaccine candidates.

## Methods

### Ethics statement

The protocol was approved by the Ethics committee of Centro Internacional de Vacunas (CECIV). Samples from volunteers were collected anonymously and not linked to the identity of the donor. Written informed consent (IC) was obtained from each volunteer at enrollment. All volunteers were adults.

### *Plasmodium vivax* gametocyte samples

**Fig 1:**
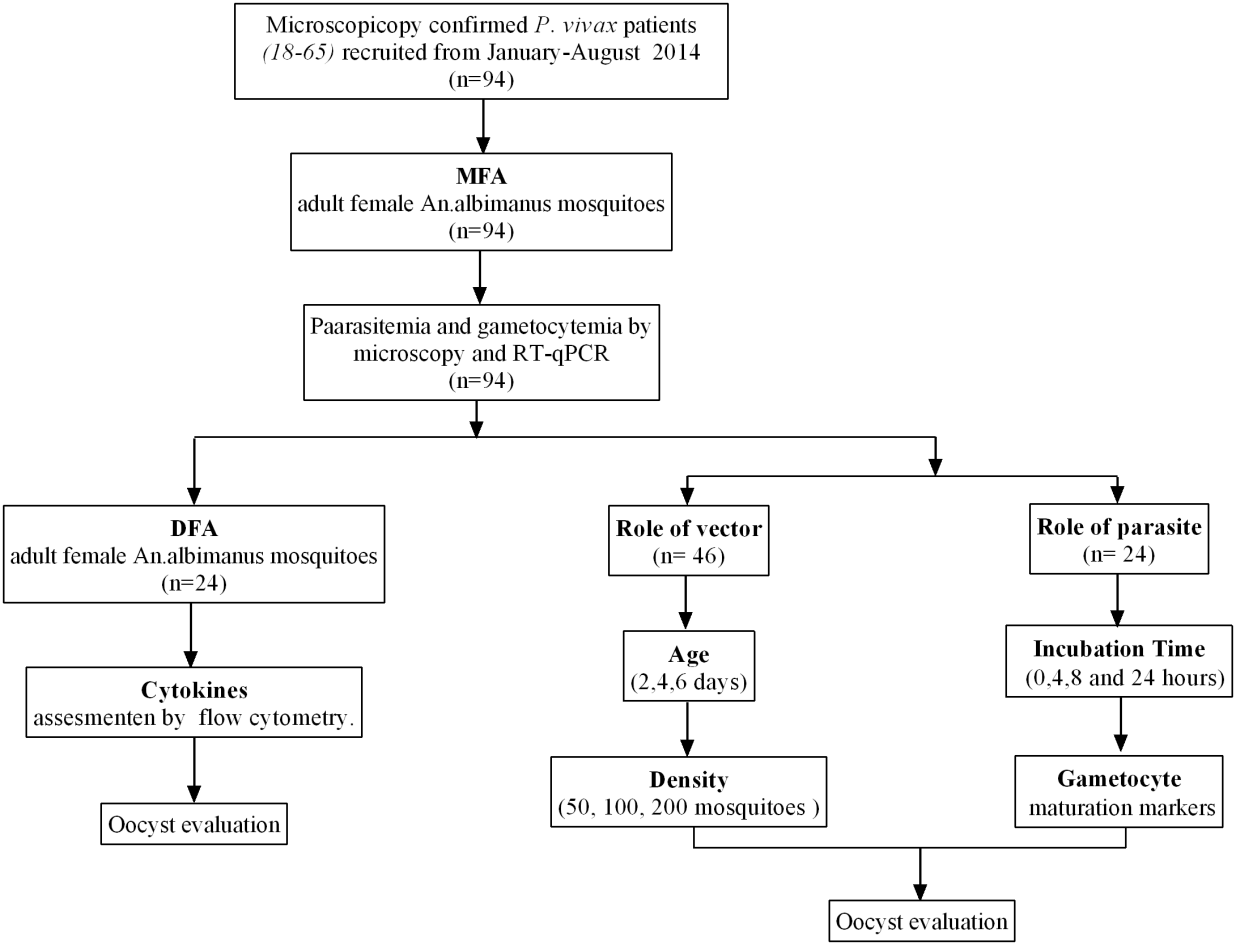
Volunteer enrollment and study scheme.

Blood samples harboring *P*. *vivax* parasites were obtained from symptomatic (fever (axillary temperature >37.5°C), malaise, chills, and/or headache) and microscopically confirmed *P*. *vivax* malaria patients who presented to malaria outpatient clinics in Tierralta, Cordoba and Buenaventura, Colombia between January and August, 2014. A total of 94 parasite donors, men and women aged 18–65, were recruited from outpatient centers and signed an IRB approved informed consent (IC) before enrollment (Fig 1). A total of 10 mL of blood was drawn from each volunteer for molecular confirmation of *Plasmodium* species, quantification of parasite and gametocyte density and maturation state, and subsequent MFA. Sera were used for cytokine level measurement. Samples were fractionated and handled as described below. A subgroup of 24 volunteers was asked for their willingness to allow mosquitoes to directly feed on the forearm (DFA).

### Malaria diagnosis

Before enrolment, malaria diagnosis was performed by microscopy and later on confirmed by qPCR. Patients were considered malaria positive and included in the study if confirmed to be positive exclusively for *P*. *vivax* infection by microscopy and reported not having initiated any anti-malarial treatment before diagnosis. For microscopic blood examination, two drops of blood (~100μL) were obtained by finger prick, deposited onto glass slides, and stained using Giemsa stain method [38, 39]. Presence of sexual and asexual parasites was independently confirmed by two experienced microscopists, using 200 leukocytes as reference for quantification. Parasitemia and gametocytemia were reported per microliter assuming an average of 8000 cells/μL [40].

### Real time quantitative PCR (qPCR)

Parasitemia was confirmed and quantified by the qPCR method using Taqman probes based on Plasmodium sp. 18S as previously described [41]. Positive control DNA, and a calibration curve of known parasitemia for *P*. *falciparum* and *P*. *vivax* were included in each run including the extraction of a negative control. Samples were considered negative if an increase in the fluorescence signal was observed after a minimum of 40 cycles.

### Direct Feeding Assay (DFA)

Volunteers who accepted direct mosquito feeding were subjected to the DFA as previously described [42] with minor modifications. Briefly, batches of 30 adult (3–4 days after emergence) female *An*. *albimanus* mosquitoes were placed in feeding boxes, starved overnight, and then fed directly on *P*. *vivax* positive volunteers for 10 minutes. Fed mosquitoes were transported in cages to secure infection rooms and kept under strict security and laboratory conditions (constant 80%, humidity and 26°C temperature). Mosquito infections were evaluated seven days after DFA by microscopic examination of midguts (DNA was extracted from 30 midguts of individual mosquitoes to determine the number of parasites present in each sample by qPCR) and oocyst counts, as well as by RT- qPCR seven days after DFA, additionally sporozoite loads in salivary glands were assessed 14 days after DFA.

### Membrane Feeding Assay (MFA)

MFA was performed in two mosquito colonies located in Buenaventura, and Tierralta two sites with local malaria transmission. Infected blood samples were sent at 37°C from endemic areas to the nearest mosquito colony. Blood samples were centrifuged at 3000 rpm to separate iRBCs from plasma, were washed twice with incomplete RPMI and subsequently reconstituted with a pool of AB^+^ sera obtained from healthy donors at the Red Cross Blood Bank (Cali, Colombia). A total of ~100 adult female *An*. *albimanus* mosquitoes (3–4 days after emergence) were subjected to overnight fasting and the next day were placed into feeding and cages (10x10x5 cm) provided with an glass feeder (Ø 3 cm) with and artificial membrane. Mosquitoes were allowed to feed for 20 minutes at 37°C after which, unfed mosquitoes were removed from the cages and fed mosquitoes were transported to secure infection rooms as described above in the DFA section. For each MFA, the amount of blood required was calculated considering that each mosquito consumes ~3.5 μL of blood per feed.

Mosquito infection was evaluated by light microscopy to assess the number of oocysts in mosquitoes midguts, after staining with 2% mercurochrome. Infection was evaluated on day seven and sporozoites in salivary glands on day 14 after MFA. Microscopic counts were compared with those of qPCR individually. First, each mosquito midgut was microscopically evaluated, then the midgut was quantitatively transferred to a micro-centrifuge tube for individual DNA extraction. DNA was extracted using the PureLink Genomic DNA kit (Invitrogen, CA) using the tissue protocol with 12 hours of proteinase K digestion. PCR was performed using Taqman probes as was described [12, 43–45].

### Influence of mosquito age and density on MFA outcomes

In order to assess the influence of mosquito age on infectivity, MFAs were performed using three different mosquito age groups: 2, 4 and 8 days after emergence. Likewise, the influence of mosquito density was tested by using mosquito groups of 50, 100, 200 and 300 per cage.

### Influence of incubation time on MFA outcomes

Additionally, the influence of the delay to perform the MFA after blood draw was tested at different time intervals: 0, 4, 8 and 24 hours after donors’ bleeding. In this case ~130 μL of blood were collected for both exflagellation and gametocyte maturation analysis and MFA were performed using four day-old mosquitos at a 100 mosquito/cage density. A total of 24 independent assays varying the incubation time were performed with different *P*. *vivax* isolates. For this part of the study the sample collection and feeding where performed at insectaries we have developed in endemic areas, in this case in Tierralta (Cordoba) at the same building of the malaria clinic. The time 0 indicates and assays performed within the first 30–45 min after blood collection.

### Gametocytes maturation assay

To study the influence of parasite sexual stages maturation on mosquito infectivity, the mRNA expression profiles of *18S*, *Pvs 25* (stage V), *Pvs16* (stage I-IV), *PvNeK* (microgametocytes) and *PvMap* (macrogametocytes) were assessed by RT-qPCR (n = 42). Whole blood samples were stored in RNA stabilizing solution (Tempus) at -20°C until analysis. The RNA was purified using affinity columns (Qiagen, Hilden, Germany) and cDNA was transcribed using Superscript III (Invitrogen, Carlsbad, CA) according to manufacturer’s instructions [46]. Transcripts were evaluated using SYBR green as previously described[47].

### Influence of host immunological factors on mosquito infection outcome

Sera collected from parasite donors were studied to determine the plasma levels of IL-2, IL-4, IL-6, IL-10, TNF-α and IFN-γ using the Cytometric Bead Array (CBA, catalog No. 551809 BD Biosciences Pharmingen, USA) (n = 24) according to manufacturer’s instructions. The infected blood samples were drawn in EDTA tubes and the plasma was aliquoted for analysis. Ten samples from non-infected volunteers were used as baseline cytokine levels. The samples were evaluated in duplicate and in a FACS Canto-II flow cytometer. Standard curves for each cytokine were generated and the concentration calculated using the BD FCAP Array Software v 1.0.1 (BD Biosciences).

#### Data management and statistical analyses

Study data were collected and managed using REDCap (Research Electronic Data Capture) secure electronic data capture tools hosted at Caucaseco Scientific Research Centre [48]. All statistical analyses were performed using Matlab 2014a software. Mosquito infection rates were analyzed by delta analysis and group differences tested by chi-square tests with statistical significance level of 5%. The Wilcoxon signed-rank test for matched pairs was used for pairwise comparisons of the different assays using blood from the same donor. The correlation between gametocyte density and mosquito infection rates was measured using Spearman’s correlation.

## Results

### Volunteers recruitment

A total of 94 *P*. *vivax* infected blood samples were collected (24 from Buenaventura and 70 from Tierralta) and distributed as follows: 70 samples to determine vector factors and 24 samples to determine parasite factors and comparing MFA *vs* DFA as part of the host factors study. Most of the participants (57.3%) were female and the population was predominantly young adults, 65.4% between 18 and 35 years of age (S1 Table).

### Mosquito factors

#### Age and density

**Fig 2:**
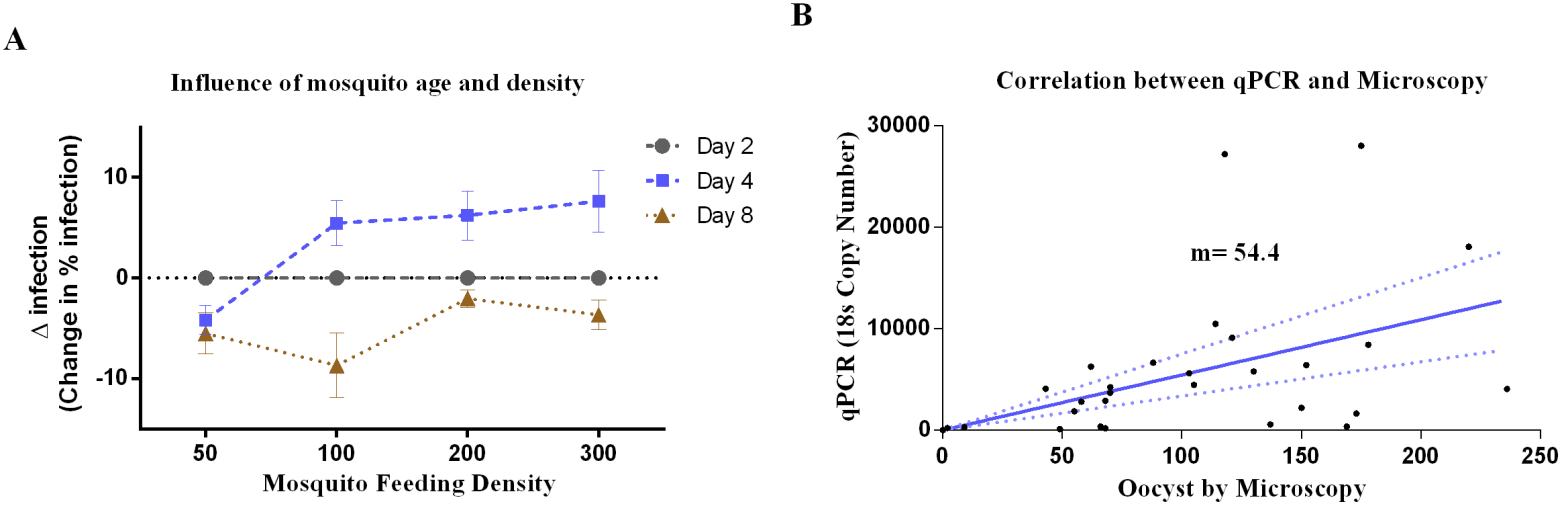
Mosquito factors affecting infectivity. **A)** Influence of mosquito age and density; mosquitoes aged 2, 4 and 8 days at the indicated densities per cage were assessed for infectivity. Data is shown as delta analysis. **B)** Correlation between PCR targeting 18s and microscopy measurement of infectivity in mosquitoes at day 7 following feeding.

The optimal mosquito age and cage density factors were evaluated using 46 assays. Four-day-old mosquitoes showed the highest infection prevalence, followed by two and eight-day-old mosquitoes (Fig 2A). The minimum mosquito density with the highest infection prevalence was 100 mosquitoes/cage. No significant differences were found between 100, 200 or 300 mosquitoes/cage, whereas 50 mosquitoes/cage density was the least efficient.

#### Oocyst quantification

To assess the mosquito *P*. *vivax* infection rates at day 7, oocysts were quantified by PCR and microscopy. Probably due to the polypoid nature of the oocysts, the number of parasites determined by qPCR was significantly higher in comparison with the number of oocysts counted by microscopy. The slope in this chart is 54.4 which could be interpreted as the average number of 18s copies per oocysts in all the tested samples, however de values ranging from 5.2 to 340. The amount of parasites obtained by qPCR presented a normal distribution and showed a proportion of 54.4 oocysts (ranging from 5.2 to 340) detected by PCR for each one detected by microscopy (Fig 2B).

### Parasite factors

#### Parasitemia and gametocytemia quantification

**Fig 3:**
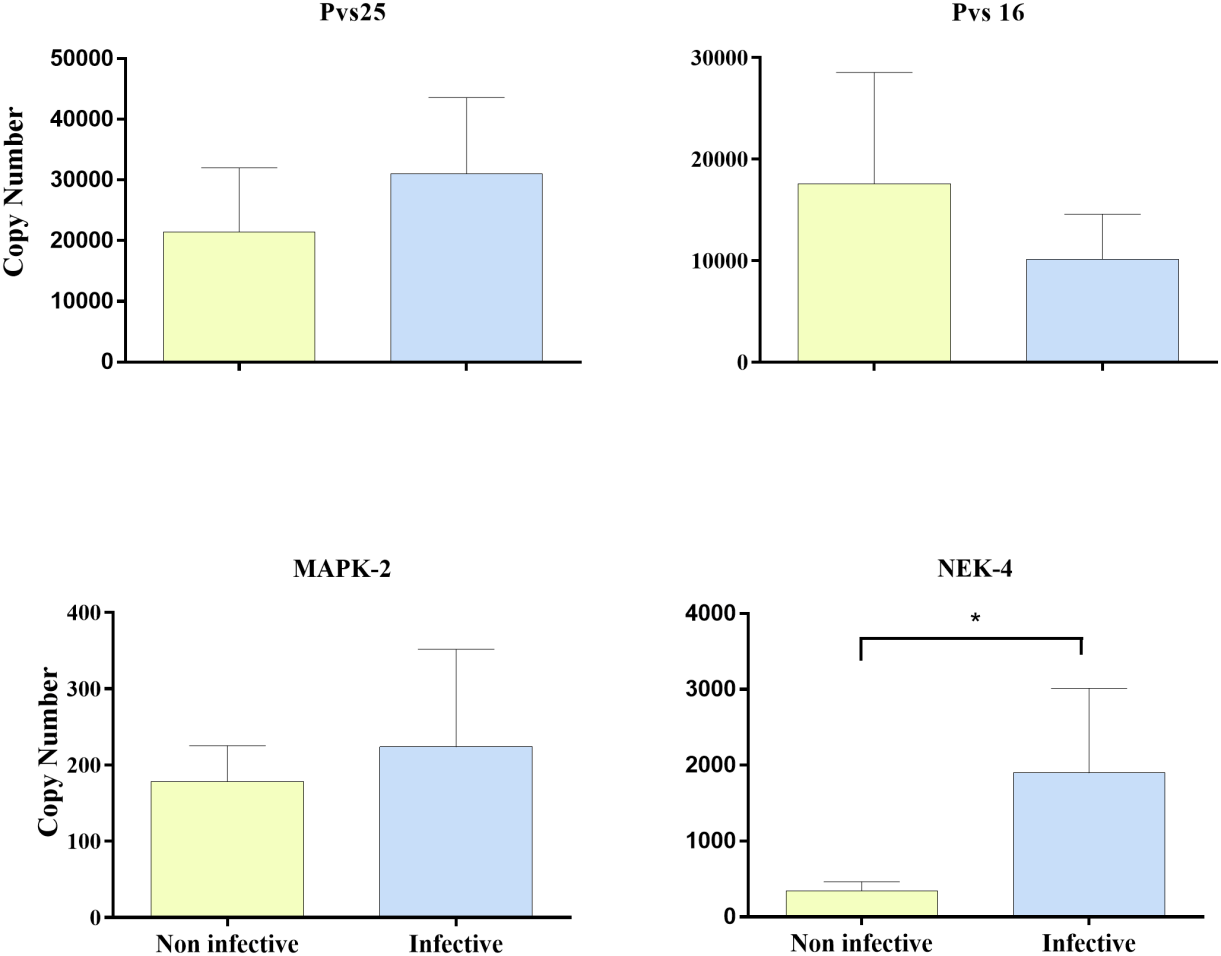
MFA. Levels of molecular markers observed by infective and non-infective samples. *Pvs25*: mature gametocytes; *Pvs16*: *immature gametocytes*; *MAK-2*: male gametocytes; *Nek-4* female gametocytes. A total of 94 infected blood samples were used to correlate *P*. *vivax* infectivity with gametocyte markers.

A total of 94 infected blood samples were used to correlate *P*. *vivax* infectivity with gametocyte quantity, maturation stage and male/female ratio by RT-qPCR. A total of 24 of these samples also were analyzed after 4, 8 and 24 hours after blood draw (Fig 1). High *Pvs25* (mature gametocytes), *Pvs16 (immature gametocytes)*, *MAK-2* (male gametocytes) and *Nek-4* (female gametocytes) transcription levels were observed in samples that were more infective in *An*. *albimanus* mosquitoes. In addition, statistically significant differences in *Nek-4* levels were observed between infective and non-infective samples (Fig 3) indicating a correlation between female gametocytes and infectivity. Although circulating gametocytes were found in all samples, infection outcome was better in samples with higher *Pvs25* (non-significant) and *Nek-4 (p = 0*.*02)* indicating that the mature gametocytes and macro and micro-gametocytes proportion play a role in parasite infectivity.

#### Incubation time assays

**Fig 4:**
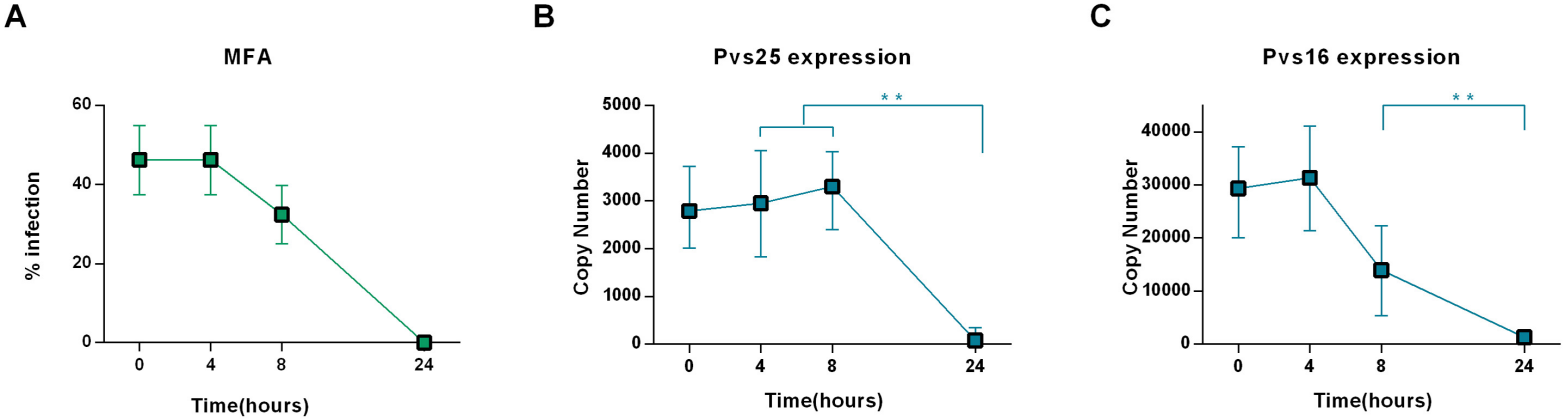
Effect of the incubation time. A) Time course of the parasite infectivity after 0, 4, 8, 24 hours of Blood draw. B) Pvs25 expression after blood draws. C) Pvs16 expression after blood draws. A total of 24 independent assays varying the incubation time were performed with different *P*. *vivax* isolates of which 11 were infective. Each data point represents the average of 11 independent assays.

A total of 42 infected blood samples were collected to assess the role of time between the blood draw and infectivity, of which 29 were infective (69%) MFA was performed at different time intervals after blood-draw (0, 4, 8 and 24h) maintaining the samples at constant temperature (37°C). In comparison with assays performed immediately after the blood draw (0h), assays performed at 4h showed highest mosquito prevalence infection and oocyst levels. The assays performed at 8h after blood-draw showed ~20% decrease in the infection rates compared with 4h. Assays performed 24h after blood-draw were entirely unsuccessful (Fig 4). Significant differences in infection rate were found between assays at 4h and 8h after blood-draw (p = 0.0018). The exflagellation response was in line with the infectivity.

#### Influence of parasite incubation on the expression of gametocyte markers

At each time interval, an aliquot of 200 μL of infected blood was stored in RNA buffer (Tempus) and kept at 4°C until processing. A subset of samples (n = 11) were analyzed to determine the effect of parasite incubation on the *Pvs16* and *Pvs25* mRNA expression. Sample infectivity and transcription levels of *Pvs25* were strongly related. *Pvs25* transcription levels were similar until 8h after blood-draw; thereafter, a drastic decrease is observed. Significant differences were found between the number of copies of *Pvs25* at 4h and 24 h (p<0.01). The Pvs25 transcription levels decreased until <100 copies at 24h after blood-draw, where they were insufficient to induce infection in mosquitoes (Fig 4).

### Host factors

#### Cytokines role

**Fig 5:**
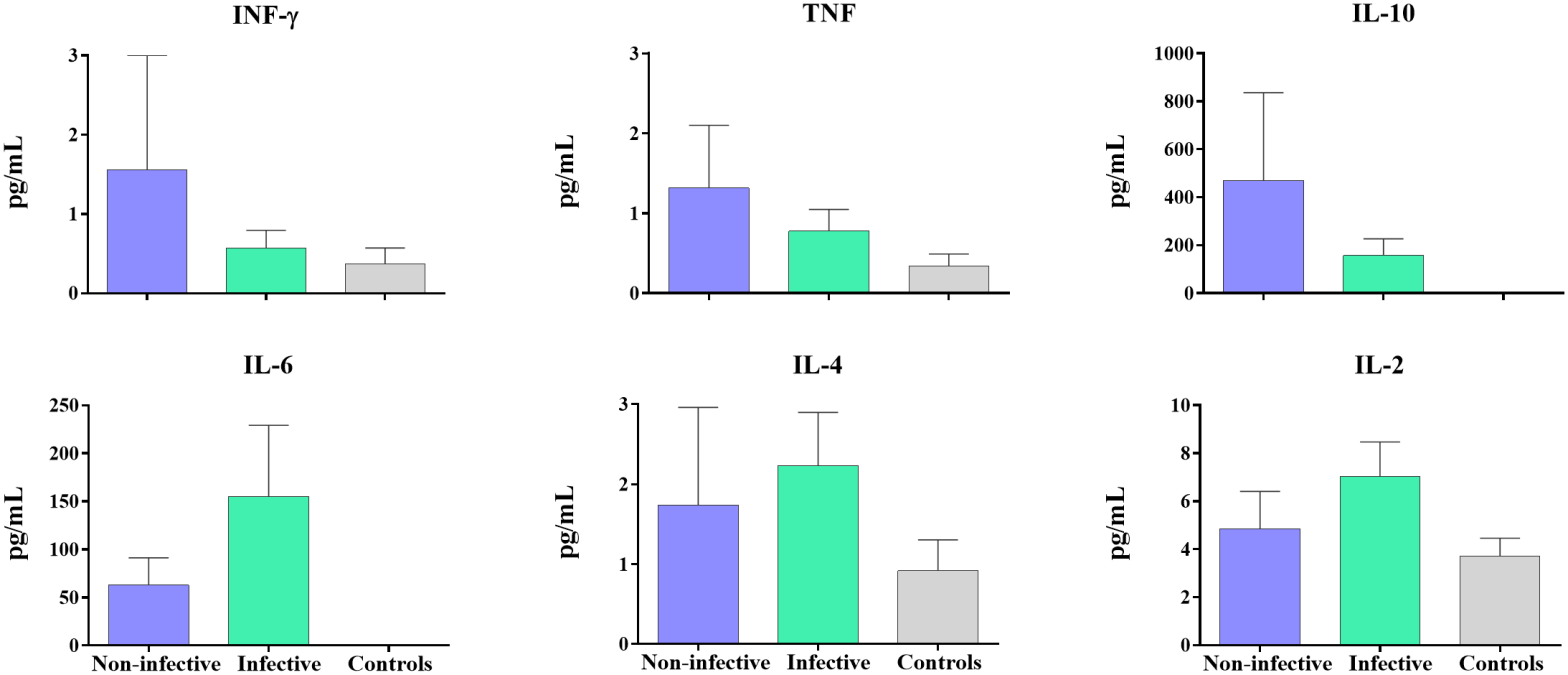
Cytokines levels (pg/mL) of 24 *P*. *vivax* volunteers. Levels of cytokines measured in *P*. *vivax-*infected volunteers bitten by 30 uninfected *An*. *albimanus* mosquitoes during DFA are shown divided in two categories: non-infective and infective to mosquitoes (median + interquartile range).

All infected volunteers displayed elevated IL-10 values as has been previously observed for *Plasmodium* infection [49, 50]. A comparative analysis was performed between mosquito infection transmitting versus non-transmitting infected volunteers, which showed a trend towards increased IFN-γ, TNF and IL-10 in patients who failed to transmit (Fig 5). The levels of the inflammatory cytokines IFN-γ, TNF α and IL-6 were found to have a negative significant correlation with *Pvs*25 (p = 0.005; 0.008; 0.01 respectively) expression and subsequently with the parasite maturation status (Fig 5). In addition a positive correlation was observed for most of the evaluated cytokines (S1 Fig)

#### Comparison of DFA and MFA

**Fig 6:**
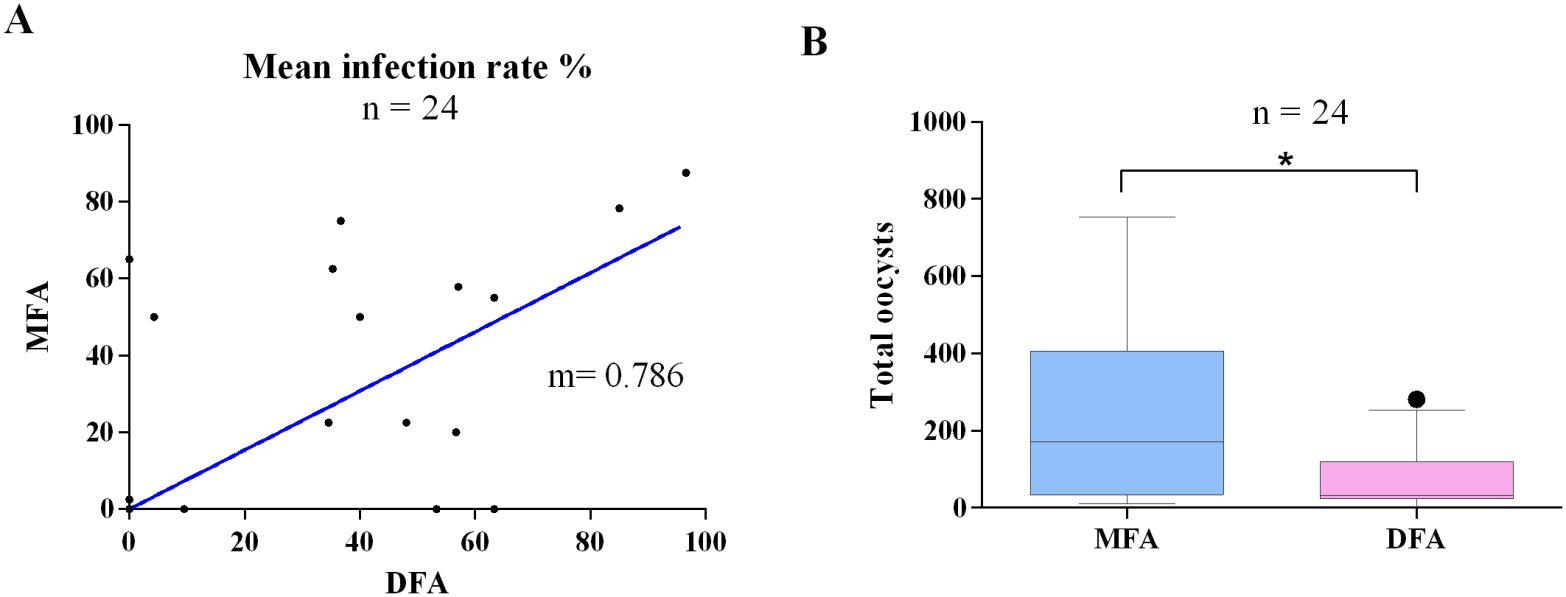
MFA and DFA infectivity comparison. A) Mosquito infection rate correlation B) Oocyst infection intensity comparison. A total of 24 independent assays were performed.

A total of 24 volunteers were enrolled to compare DFA and MFA infectivity. The two types of assays were carried out simultaneously. More than half [54% (13/24)] of samples infected mosquitoes at rates >40%. A higher median infectivity rate was observed in DFA compared to MFA. However, the difference was not statistically significant. In contrast, MFA showed a statistically significant higher oocyst count compared to DFA (p-value = 0.048) (Fig 6). A total of four samples presented wide differences in infectivity results in both techniques. One sample was infective in MFA but negative in DFA. This sample has high levels of TNF (4.3 pg/mL) and IL10 (379 pg/mL). The other three samples were negative by MFA and positives in DFA. In these samples high levels of Il-6 were observed (233 pg/mL as mean).

## Discussion

In this study we found that human, parasite and mosquito factors (i.e. cytokine levels, gametocyte markers, time between the blood draw) play a fundamental role in *P*. *vivax* infectivity, thereby affecting the MFA outcome. In contrast to other vaccine candidates where the vaccine efficacy is tested *in vivo*, TB vaccine efficacy relies on the MFA to assess the ability of immune responses to reduce or block parasite transmission to the mosquito. Whereas with *P*. *falciparum* MFA can be easily performed with cultures of well-characterized parasite strains/clones, for *P*. *vivax* these assays are more labor intensive as they rely on the availability of parasite infected blood from acutely infected patients, introducing significant variability. In this study a total of 94 *P*. *vivax* field isolates were used to optimize an assay to evaluate TB immunity.

High levels of the regulatory cytokines IL-10, IFN-γ and TNF were correlated with low parasite infectivity. This T-cell immune response explains the infectivity outcome in the study groups. According to previous studies, fever/chills have a deleterious effect on the parasite, most likely associated with cytokine release. The gametocyte density, measured as *Pvs25* gene expression, was highly related with the parasite infectivity. In spite all the samples were positives for Pvs25, the samples with high copy numbers were the most infectious, furthermore, statistically significant differences were observed with PvsNek-4, indicating that the gametocyte density and maturation status are correlated with parasite infectivity. We found a negative correlation between the levels of IFN-γ, IL-10 and TNF and the *Pvs25* expression, indicating that the immune response may have a bigger impact on the viability of gametocytes.

The oocyst determination by using qPCR showed a high proportion of 18S DNA copies detected for each oocyst showing the multinucleated nature of the oocysts. In addition, the oocyst size, ranging between 5–50 μm [51], could be able to carry high numbers of haploid sporozoites before the rupture. Interestingly the use of PCR presents the infection load as potential sporozoites released, which could be a more accurate measure of the transmission potential.

The maximum infectivity level was achieved 4 hours after blood-draw, coinciding with increased expression of *Pvs25*.Of the total samples that infected mosquitoes, only 31% showed a similar infection rate and total oocyst count at 4h and 8h. This could be due to gametocyte maturation boosted by stress and nutrients decrease at 8h after blood drawn. These results indicate that parasite infectivity is time-limited and supports the use of TB assays (TBAs) performed no later than 4 hours after blood draw in order to obtain reliable results. This requires the laboratory mosquito colony be within 4 hours travel-time from the endemic areas.

Infections of *Anopheles* mosquitoes by DFA are considered the gold standard for *P*. *vivax* infectivity due to the lack of *in vitro* culture. However, most of the reported studies have been performed using only MFA. In this study we assessed parasite infectivity using both to investigate the influence of human factors. We found no significant differences between DFA and MFA mosquito infection rate. However, oocyst intensity was significantly higher for MFA, indicating a possible role of cytokines in parasite maturation at the mosquito level. Even though the high correlation between DFA and MFA would suggest that either of the two techniques can be used to measure TB, they also have applications for which one would be better than the other. In general terms DFA would better indicate TB in the host i.e. the parasite transmissibility in the presence of antibodies, cytokines and human host cells. On the other hand, MFA would be highly complementary in allowing a separate assessment of specific TB responses, and parasite factors associated with transmission i.e. parasite maturation, role of macro and microgametocyte density and proportion, parasite diversity and others[12].

We optimized the variables to develop an assay to assess TB immunity against *P*. *vivax*. This assay could be used for implementation of TBA in appropriate regions of Latin America and to assess the potential of current TB vaccine candidates.

## Supporting Information

S1 Table Volunteer enrollment breakdown. (XLSX)

S1 Fig Cytokines correlation. (PNG)
