# Supplementary figures and images for: Optimization of a Membrane Feeding Assay for Plasmodium vivax Infection in Anopheles albimanus
Source: PLoS Negl Trop Dis. 2016 Jun 29;10(6):e0004807. doi: 10.1371/journal.pntd.0004807 (PMC4927173; doi:10.1371/journal.pntd.0004807)

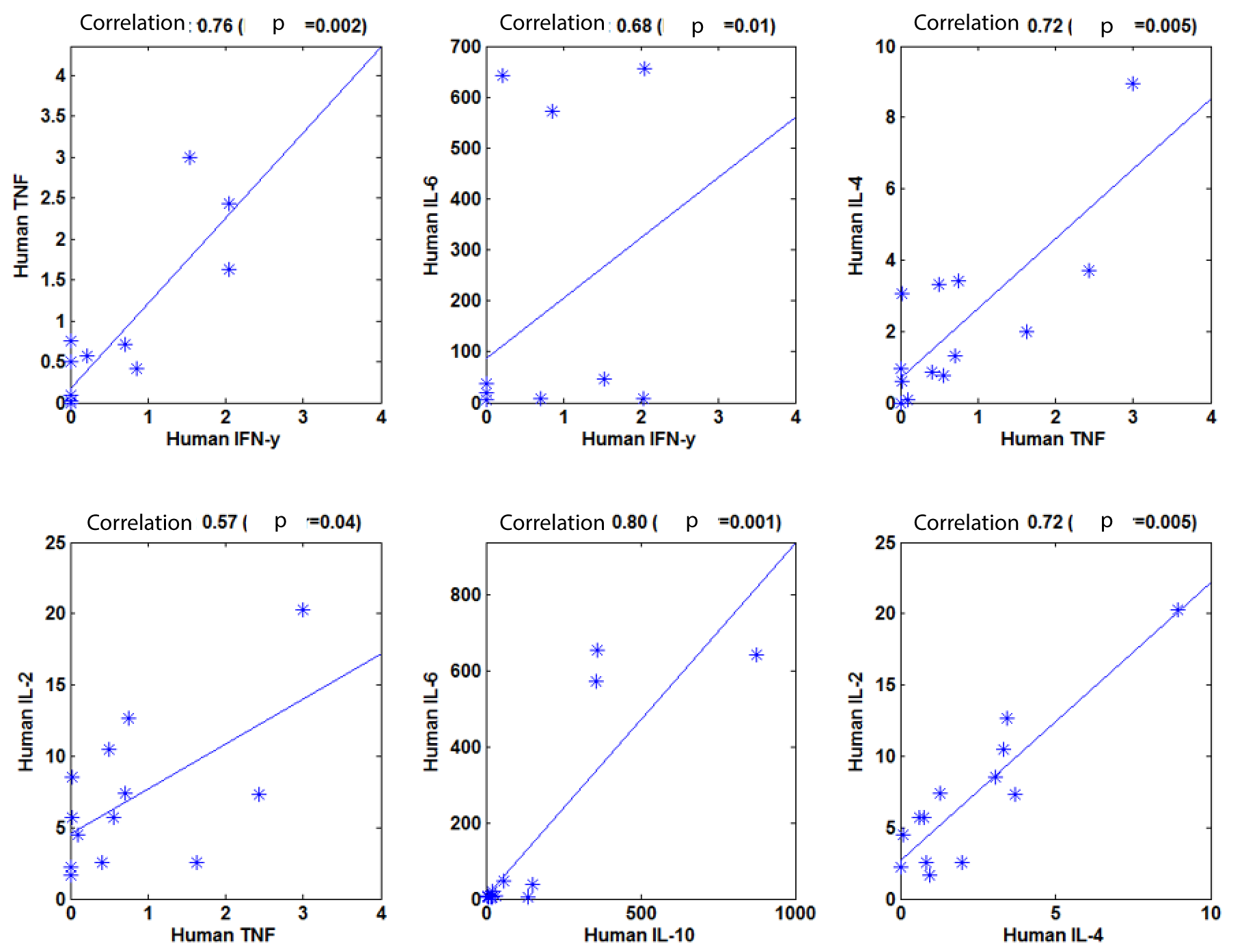

Supplement: S1 Fig — (PNG) [file pntd.0004807.s002.png]
